# Influence of aged and non-aged environmentally relevant microplastics on zinc removal from aqueous media by *Daphnia magna*

**DOI:** 10.1007/s00216-026-06592-1

**Published:** 2026-06-12

**Authors:** Mary Isabel Villacís, Laura Torrent, Mònica Iglesias, Emili Besalú, Laura Borgese, Stefania Federici, Eva Marguí

**Affiliations:** 1 Department of Chemistry, Universitat de Girona, Girona, Spain; 2 Department of Mechanical and Industrial Engineering, University of Brescia and INSTM, Brescia, Italy; 3 Institute for Water Research (ICRA- CERCA), Science and Technology Park of the University of Girona, Girona, Spain

**Keywords:** *Daphnia magna*, Zinc, Microplastics, Tertiary wastewater treatment, Nature-based solution

## Abstract

Recent studies have highlighted the potential of *Daphnia magna* (*D. magna*) as a bio-based tertiary system for improving treated water quality. However, further research is needed to study the impact of micropollutants on these planktonic crustaceans. The present work provides an in-depth investigation of how microplastics (MPs), emerging contaminants of high-environmental interest, can influence the removal of Zn^2+^ (selected as a pollutant model) from water by *D. magna* individuals. With this purpose, true-to-life MPs were obtained by micronizing commercially available plastic devices of different polymers (polyethylene, polypropylene, and polystyrene) and sizes (<63 µm and 63–80 µm). Additionally, the produced MPs were exposed to UV radiation to simulate aging processes in natural environments that can lead to changes in their physicochemical properties, and their bioavailability and toxic effects to organisms. An integrated analytical framework combining complementary techniques was employed, including confocal microscopy to detect and localize MPs in *D. magna* individuals, infrared spectroscopy to characterize structural changes in aged and non-aged MPs, and inductively coupled plasma optical emission spectroscopy (ICP-OES) for quantitative determination of zinc in aqueous samples and digested organisms. Results showed that although the presence of MPs does not significantly affect Zn^2+^ removal from the aqueous media, MP polymer type or aging state affects Zn^2+^ accumulation in the living organisms. On the contrary, the effect of MP size seems to be negligible, at least in the studied size range (<63 versus 63–80 µm).

**Graphical abstract:**

**Figure:**
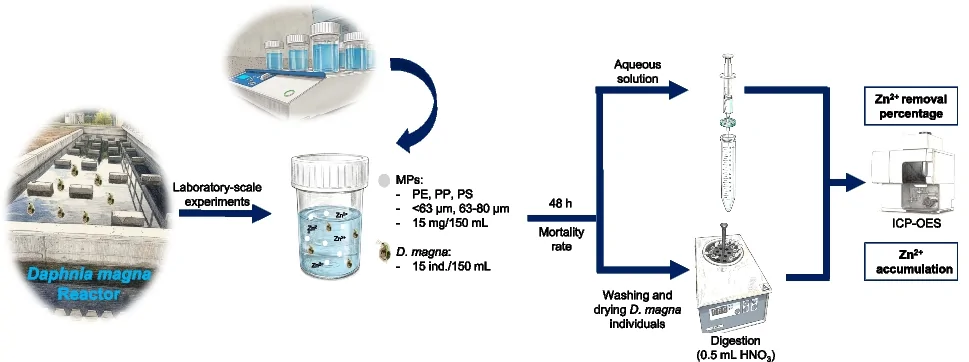

**Supplementary Information:**

The online version contains supplementary material available at https://doi.org/10.1007/s00216-026-06592-1.

## Introduction

Water scarcity is a critical issue in numerous areas worldwide, especially in semi-arid and arid Mediterranean regions, where irregular precipitation fails to meet water demands. Climate change worsens the situation, negatively impacting drinking water resources and crop production [1, 2]. To address these challenges, water reuse has been advocated by various executive bodies and intergovernmental organizations. For instance, a primary objective (Target 6.3) of the United Nations’ sixth Sustainable Development Goal (SDG 6) is to achieve a global increase in water recycling and safe reuse by 2030 [3–5].

Treated wastewater represents the most accessible and reliably available resource for water reuse applications [3]. The new directive of the European Parliament and of the Council concerning urban wastewater treatment proposes the incorporation of advanced treatments in wastewater treatment plants to eliminate the broadest possible spectrum of micropollutants with the aim to decrease their presence in the receiving waters and their potential uptake by plants and soils when reclaimed water is reused for crop irrigation [6]. Nature-based solutions are emerging as sustainable alternatives to traditional wastewater treatments because they eliminate dependency on chemicals, require less energy, and minimize environmental impact while optimizing operational costs [7, 8, 8]. Nature-based solutions that mimic natural remediation processes using solar energy and living organisms, such as *Daphnia magna* (*D. magna*), are particularly interesting. *D*. *magna* is a large planktonic crustacean belonging to the order Cladocera, with a diet consisting of particles smaller than 50 µm in diameter, including algae, organic detritus, protists, and bacteria. These living organisms can adapt to environmental changes in a short timeframe due to their significant capacity for both genetic adaptation and phenotypic plasticity [9, 10]. A key attribute is their ability to reproduce rapidly via parthenogenesis, which is beneficial for maintaining the high population densities required for large-scale water treatment. Beyond removing inorganic and organic suspended particles, the feeding activity of *D. magna* helps to reduce bacterial populations, such as *Escherichia coli* and coliforms, while also lowering biological oxygen demand (BOD) through the consumption of particulate organic matter [8] . However, *D. magna* is sensitive to relatively high concentrations of common contaminants found in raw wastewater and therefore, its use in the field of water treatment technology is mostly limited to tertiary/quaternary treatment stages. Several studies conducted in the last few years demonstrated the potential of *D. magna* as an effective innovative nature-based tertiary treatment [11, 12]. Even so, further research is needed to investigate the elimination and potential effects of micropollutants on these planktonic crustaceans, including microplastics (MPs). Since these particulate contaminants in the micrometric size range can be ingested by *D. magna* [13], it is reasonable to expect that their presence may influence the ability of these organisms to remove other contaminants from water. For example, the uptake of metals by *D. magna* from aquatic systems has already been demonstrated [14], but there is still limited information on the removal of these contaminants when MPs are present in the aquatic environment. It is important to highlight that most published studies addressing *D. magna* and MPs have primarily focused on toxicological effects, rather than their influence on metal removal [15, 16].

The present work provides an in-depth investigation of how MPs with different characteristics (i.e., polymer types (polyethylene (PE), polypropylene (PP), and polystyrene (PS)), particle sizes (< 63 µm and 63–80 µm), and aging processes) can influence the capacity of *D. magna* to remove Zn^2+^ (as a pollutant model) from water under laboratory-scale conditions. A comprehensive analytical approach based on complementary techniques was applied for such purpose. Confocal microscopy was used to identify and localize MPs within *D. magna* individuals, infrared spectroscopy was employed to assess structural differences between aged and non-aged MPs, and inductively coupled plasma optical emission spectroscopy (ICP-OES) was used for the estimation of Zn^2+^ removal in the aqueous solution with and without the presence of MPs. In addition, to study the possible effects on *D. magna* individuals, the survival rate and the bioaccumulation of Zn^2+^ in living organisms at the end of the experimental tests were also considered.

Results derived from this work are of paramount interest to get additional knowledge about the potential of *D. magna*–based technology as an eco-sustainable tertiary/quaternary wastewater treatment system for micropollutants removal.

## Materials and methods

### Reagents and materials

A 1000 mg·L^−1^ zinc stock solution (in HNO_3_ 0.5 M, ROMIL PrimAg, UK) was used for preparing aqueous solutions to assess the metal removal efficiency by *D. magna* from water. Tween 80 surfactant, acquired from Sigma-Aldrich (Germany), was used for preparing MP suspensions. Ultrapure water was obtained from Millipore Milli-Q Plus System (Germany, 18.2 MΩ·cm^−1^ at 25 °C and < 5 µg·L^−1^ total organic carbon). Nitric acid (69%, Panreac, Spain) was employed for digesting *D. magna* individuals.

### Microplastics

PE “UVPMS-BV-1.00” fluorescent MPs (53–63 µm, Cospheric, USA) were employed in some of the experimental tests. To better simulate the MPs that can be found in real scenarios, PE, PP, and PS polymers were also prepared from daily life utensils (true-to-life MPs) and used in laboratory experiments. The original plastic macropieces were fragmented through a mechanical process using a ultracentrifugal mill (Retsch ZM 200) equipped with a cyclone connected to a vacuum cleaner. After, MPs of different size ranges (< 63 µm, 63–80 µm) were obtained by sieving the milled plastic fragments through a set of appropriate meshes. Specific details on the preparation protocol are described elsewhere [17, 18]. As mentioned in the “Introduction” section, MPs in the natural environment can undergo aging processes that can lead to changes in their surface and adsorption capacities. To assess it, the same true-to-life MPs were also submitted to an aging process with UV light (8 weeks at 42 °C) to simulate the effect of sunlight on MPs once they have entered the aquatic environment. Briefly, the artificial photoaging was carried out in a controlled UV chamber equipped with UVC lamps (main emission at 254 nm), operating at constant temperature (42 °C) under atmospheric conditions. The irradiance inside the chamber was approximately 40 W·m⁻^2^, ensuring accelerated photo-oxidation of the polymer surfaces. The exposure time (up to 8 weeks) was selected to induce measurable chemical and morphological changes while maintaining comparability with environmentally relevant aging stages. The UV treatment promotes oxidation processes, leading to the formation of oxygen-containing functional groups (e.g., carbonyl and hydroxyl groups) and surface modifications. A detailed description of the UV aging setup, including spectral characterization, irradiance mapping, and comparison with natural sunlight exposure, is provided in our previous work [17], to which the reader is referred for comprehensive methodological information.

### Water samples

To evaluate the influence of the characteristics of the water matrix on the filtration capacity of *D. magna*, two types of waters were considered for laboratory-controlled experiments: mineral water and wastewater from a pilot scale system designed for a biological treatment system (based on *D. magna* activity) [12]. Physical-chemical characterization of these waters is presented in the Electronic Supplementary Material (see Table S1). Specifically, Zn^2+^ content was measured to quantify the amount of this metal in the waters under study.

### Daphnia magna culture conditions

*D. magna* individuals were obtained from laboratory cultures that have been maintained for 1 year, in two 10-L tanks filled with calcium-rich mineral water (40 mg·L^−1^). The water temperature was kept at 22.0 ± 2 °C, with natural photoperiods of 12 h of light and 12 h of darkness, and a continuous air supply to prevent anoxia. The organisms were fed with a mixture of dry spirulina powder (100% *Spirulina platensis*, KeyPharm, Belgium) and baker’s yeast (*Saccharomyces cerevisiae*, Mondelez International, Spain) prepared in 1 L of bottled mineral water. The mixture was then stirred and allowed to rest for 30 min to ensure thorough homogenization and that the larger spirulina powder particles could settle. Typically, the organisms were fed twice a week. Additionally, aquarium cleaning was performed once a month, replacing one-third of the water with fresh water to ensure proper hygiene and optimal development of the organisms [19].

### Laboratory experiments setup

An aliquot of 150 mL of the solution, containing Zn^2+^ and 0.05% of Tween 80, was placed into each container, and 15 mg of MP was added if the evaluation of the influence of this contaminant on the metal filtration efficiency of *D. magna* was intended. The mixture was maintained under magnetic stirring for 24 h. After this period, stirring was stopped, and 15 *D. magna* individuals were introduced into each container (1 *D. magna* individual per 10 mL), allowing them to remain in contact with the solution for 48 h. Exposure experiments were set at 48 h to align with the 2-day hydraulic retention time of the *Daphnia* filter reactor (2 days). All experiments were performed in triplicate. In Table 1, a summary of the different laboratory tests carried out is shown. As can be seen, a systematic study of the variables that can affect *D. magna* metal removal capacity was performed. The impact of the contaminants on *D. magn*a survival was assessed by employing the OECD procedure (OECD [20]. The counting of alive *D. magna* individuals was performed at 24 and 48 h, following the OECD acute immobilization test protocol, which defines an individual as dead if it remains immobile for 15 s after gentle agitation. After 48 h, *D. magna* individuals were removed, and water samples were filtered using a 0.45-µm cellulose acetate filter for determining the content of Zn^2+^ by ICP-OES. The initial mineral water and wastewater samples spiked with Zn^2+^ were also analyzed by ICP-OES prior to the experiments listed in Table 1. Metal removal efficiency was calculated as percentage using initial ([Zn^2+]^_I_) and final ([Zn^2+^]_F_) metal concentrations.

**Table 1:** Summary of the main conditions of the different laboratory experiments with *D. magna*

**Experiment**	**Zn**^**2+**^** concentration (mg·L**^**−1**^**)**	**Surfactant (%)**	**Microplastic**				**Water type**
			**Type**	**Source**	**Size (µm)**	**Aged**	
Control	0	0	-	-	-	-	MW
							WW
1	0.25	0.1	PE	C	53–63	No	MW
2	0.25	0.05	PE	C	53–63	No	MW
			-	-	-	-	
3	0.50	0.05	PE	C	53–63	No*	MW
			-	-	-	-	
4	1.00	0.05	PE	C	53–63	No	MW
			-	-	-	-	
5	0.50	0.05	PE	TL	63–80	No*	MW
6	0.50	0.05	PE	TL	63–80	Yes*	MW
7	0.50	0.05	PP	TL	63–80	No*	MW
8	0.50	0.05	PP	TL	< 63	No*	MW
9	0.50	0.05	PP	TL	63–80	Yes*	MW
10	0.50	0.05	PS	TL	63–80	No*	MW
11	0.50	0.05	PS	TL	63–80	Yes*	MW
12	0.50	0.05	PP	TL	63–80	No*	WW
13	0.50	0.05	PP	TL	63–80	Yes*	WW

*The same experiment without the presence of *D. magna* individuals was also carried out in order to study the potential Zn^2+^ adsorption onto MPs. The adsorption of metal onto MPs was estimated using the same formula used to evaluate removal efficiency (%)

Nomenclature: *PE*, polyethylene; *PP*, polypropylene; *PS*, polystyrene; *C*, commercial; *TL*, true-to-life; *MW*, mineral water; *WW*, wastewater

$$Removal\;efficiency\;(\%)\:=\:({\lbrack{Zn}^{2+}\rbrack}_I\;-{\;\lbrack{Zn}^{2+}\rbrack}_F)/{\lbrack{Zn}^{2+}\rbrack}_{I\;}\times\;100$$

In addition to water analysis, *D. magna* individuals were collected to determine the metal accumulation within their bodies. They were removed from the aqueous medium using a Pasteur pipette, washed with Milli-Q water, and dried in the oven overnight at 60 °C. Once *D. magna* individuals were dried, they were digested at 60 °C with 0.5 mL of concentrated HNO_3_ (69%) by employing a block heater (SELECTA, Spain). The resulting digests were analyzed by ICP-OES*.* This comprehensive experimental approach allows for a thorough evaluation of Zn^2+^ removal capacity by examining simultaneously *D. magna* survival and Zn^2+^ concentrations both in the aqueous medium and accumulated within the organisms.

As shown in Table 1, the experiments marked with (*) were also conducted in the absence of *D. magna* to evaluate potential Zn^2+^ adsorption onto MPs and to assess how this process may influence Zn^2+^ removal by *D. magna*.

### Instrumentation

A Nikon A1R confocal microscope (Nikon, Japan) was used to visualize MPs inside *D. magna* individuals. The operating conditions employed were the following: 10× magnification, excitation wavelength of 543 nm, and emission wavelength of 570–620 nm. Zn^2+^ concentrations in both the aqueous medium and inside *D. magna* individuals were determined by ICP-OES (ICP-OES 5100, Agilent Technologies, Japan). The instrumental characteristics and measuring conditions used were the following: 1200 W RF power, 12 L·min^−1^ plasma flow, Zn atomic emission wavelength of 213.857 nm, axial plasma view, concentric nebulizer (sea spray), and multichannel CCD detector. To monitor the effects of the aging process on the chemical structure of the MPs, a PerkinElmer® Frontier attenuated total reflection Fourier transform infrared (ATR-FTIR) spectrometer was used. IR spectra were recorded at a resolution of 1 cm^−1^, with 32 scans over a wavenumber range of 650–4000 cm^−1^. Three replicates were made from each MP sample.

### Statistical analysis

Statistical tests were conducted to compare the mean values obtained from the different treatments (means and standard deviations were obtained from three replicates). First, an *F*-test determined if the two associated variances were equal or not. Then, according to that, comparisons were made by means of a Student’s *t*-test of two queues (null hypothesis was that the two compared means are equal) and the significance threshold value was set to *p* = 0.05. All the results were obtained using SPSS (IBM SPSS Statistics, version 29) and Minitab (Minitab Inc., Statistical Software, release 14) packages.

## Results and discussion

### Preliminary studies

#### Effect of surfactant concentration on Daphnia magna

Surfactants are chemical compounds capable of reducing the surface tension. This property allows them to facilitate the uniform distribution or dispersion of substances that normally do not mix, improving texture and consistency [21, 21]. In this study, Tween 80 commercial surfactant was used to obtain homogeneous suspensions in experiments involving MPs. This surfactant, also known as polysorbate 80, is non-ionic with a hydrophilic part (polyoxyethylene group) and a hydrophobic part (oleic acid) and has a density of 1.06 g·mL^−1^ at 25 °C. The optimal concentration was assessed to achieve a homogeneous MP suspension while minimizing potential harmful effects on *D. magna*. Preliminary tests demonstrated that the minimum surfactant concentration needed to obtain a homogeneous PE MP (53–63 µm) suspension was 0.05%. The removal efficiency of Zn^2+^ by organisms in the presence of MPs was significantly lower with increasing the surfactant concentration in the aqueous medium (*t*-test, *p*-value = 0.029). Additionally, *D. magna* mortality was higher after 48 h of exposure to a surfactant concentration of 0.1% (Experiment 1, Table 1) compared to 0.05% (Experiment 2, Table 1). In view of these results, a surfactant concentration of 0.05% was used in subsequent experiments performed with different MP materials (PE, PS, PP) and size ranges (< 63 µm, 53–63 µm, and 63–80 µm), as shown in Table 1.

#### Verification of microplastics ingestion by Daphnia magna

**Fig. 1:**
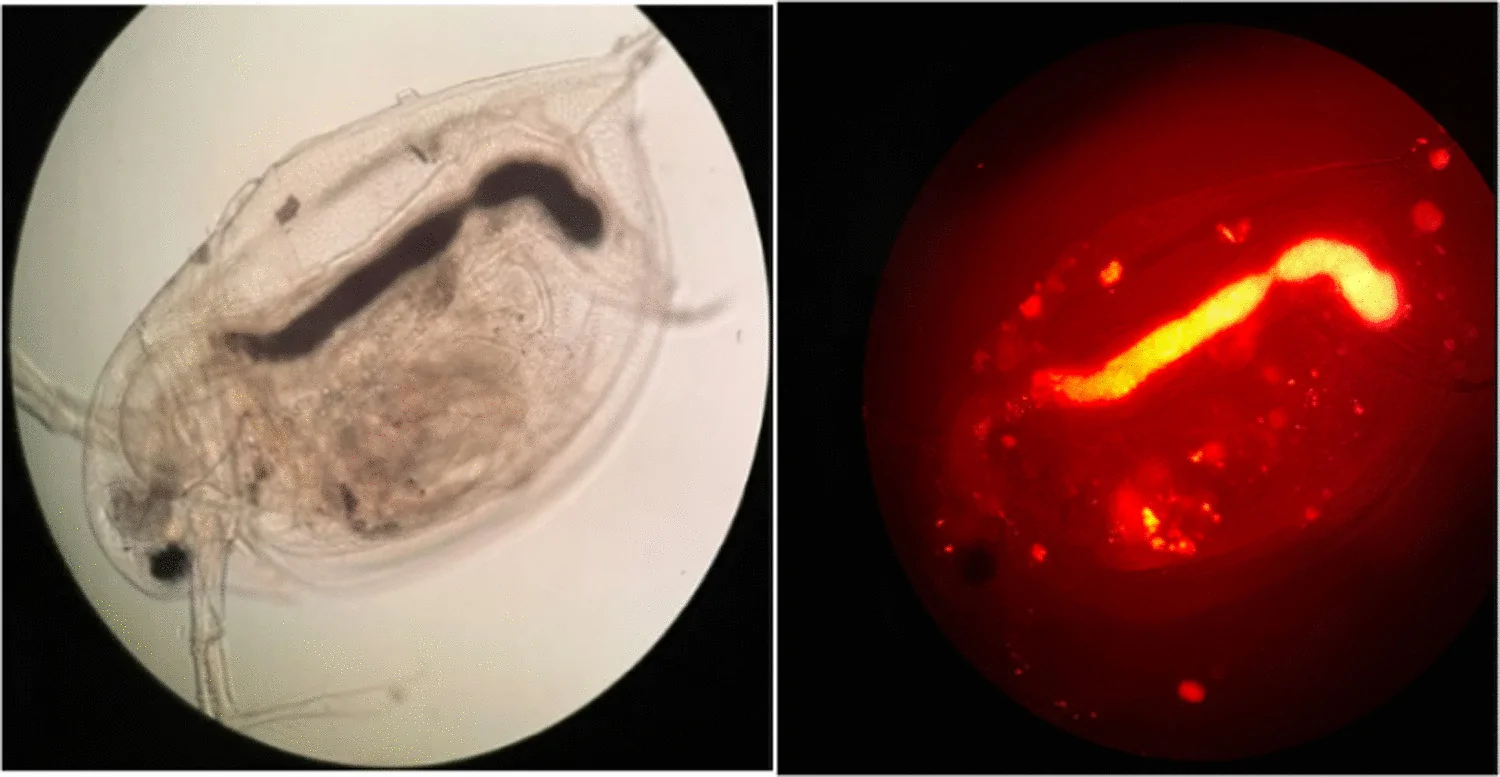
Confocal microscope images of a* D*. *magna* individual exposed to fluorescence MPs (PE, 53–63 µm) in transmitted light (left) and in fluorescence light (right). Measurement conditions are reported in section “Laboratory experiments setup”

As mentioned in the “Introduction,” it has been reported in the literature that *D. magna* individuals can ingest solid particles (< 50 μm diameter) present in water [8, 8]. An experimental test was conducted to investigate the capacity of these organisms to accumulate and eliminate MPs that can be found in the aquatic environment. For that, 15 mg of fluorescent PE MPs (53–63 µm) was mixed with 150 mL of Milli-Q water containing surfactant at 0.05% concentration. Fifteen *D. magna* individuals were added to this medium, and they were left for 48 h. After this period, one *D. magna* individual was collected from the culture medium, and it was examined using a confocal microscope. From the image obtained, it can be seen that MPs were predominantly accumulated in *D. magna*’s intestinal tract (see Fig. 1). Thus, the experiment demonstrated that these organisms could ingest MPs, and as a consequence, eliminate this kind of micropollutant from the aqueous media. Similar results were obtained by Gong et al.; however, in that work, the authors focused on the transfer of MPs from zooplankton to fish [22].

#### Effect of Zn^2+^ concentration

To evaluate the *D. magna* capacity to remove Zn^2+^, present in different concentrations in aqueous media, six experiments were conducted. Fifteen *D. magna* individuals were exposed during 48 h to spiked mineral water containing surfactant (0.05%) and Zn^2+^ at different concentrations (0.25 mg·L^−1^, 0.5 mg·L^−1^, 1.0 mg·L^−1^) in the presence and absence of commercial PE MPs (Experiments 2–4, Table 1). A control without both Zn^2+^ and MPs was also conducted for comparison purposes (Control, MW, Table 1).

**Fig. 2:**
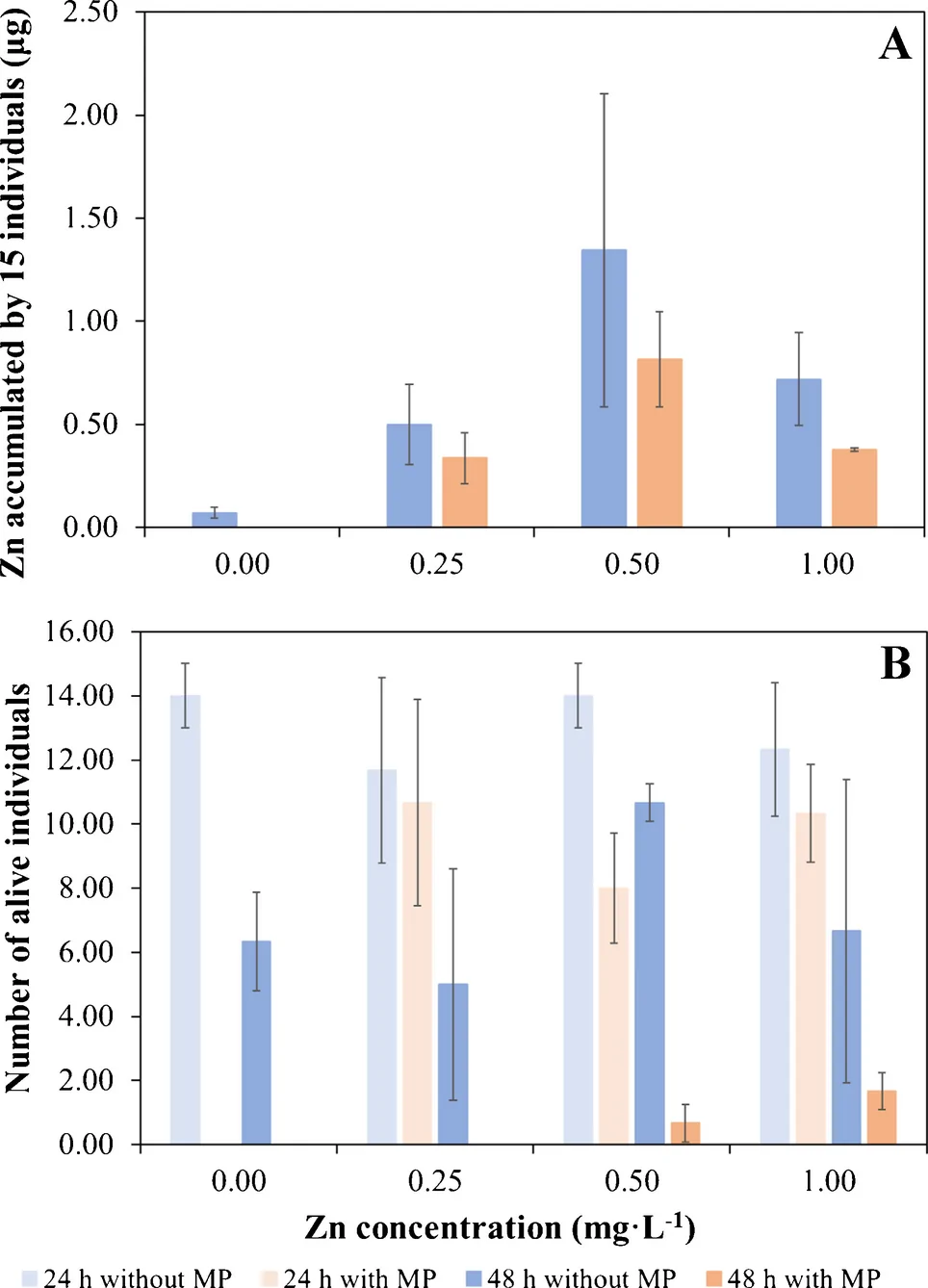
Zn^2+^ accumulated in *Daphnia magna* individuals (**A**) and *Daphnia magna* individuals’ survival (**B**). Experimental details can be found in Table 1 (Experiments Control, 2, 3, and 4). Results are expressed as mean values of three independent replicates with the associated standard deviation as error bars

As can be seen in Table 2, Zn^2+^removal efficiency was increasing by increasing the Zn^2+^ concentration in the aqueous medium. However, no significant statistical differences were observed between the presence and absence of MPs, except for the lowest Zn^2+^ concentration (*t*-test, *p*-value = 0.0035). Therefore, the experiment suggests that the presence of MPs has a minimal impact on the organisms’ capacity to remove Zn^2+^. The same trend can also be seen in Fig. 2A, where the Zn^2+^ accumulated in *D. magna* individuals is shown. At a 95% confidence level, no significant differences were observed in metal accumulation within the organisms with and without MPs (*t-*test, *p* > 0.05), with the exception of those exposed to 1 mg·L^−1^ of Zn^2+^. The impact of MPs on the ability of aquatic organisms to eliminate metals has been previously explored [23], suggesting that MPs may act as vectors for metal accumulation, potentially increasing bioaccumulation in organisms such as *D. magna*. Nevertheless, in this test, it is interesting to bear in mind that the dispersion in the Zn^2+^ content determined in *D. magna* individuals from the three experiments carried out is high due to the inherent variability of *D. magna* individuals and their low weight. This fact hampers the possibility of observing significant differences among the experimental conditions tested.

**Table 2:** *D. magna* Zn^2+^ removal efficiency from the mineral water containing different metal concentrations. Results expressed as mean ± standard deviation (*n* = 3)

	**Zn**^**2+**^** removal efficiency (%)**	
**Zn**^**2+**^** concentration (mg·L**^**−1**^**)**	**Without MP**	With PE MP (63–53 µm)*
0.25	43 ± 7	27 ± 15
0.50	76 ± 2	76 ± 3
1.00	85 ± 3	87 ± 1

*MP concentration: 100 mg·L^−1^ (15 mg/150 mL)

Nomenclature: *MP*, microplastic; *PE MP*, polyethylene microplastic

Some studies have indicated that the presence of MPs can alter the behavior and physiology of organisms, which may affect their efficiency in eliminating contaminants [24, 25]. As is shown in Fig. 2B, the mortality of *D. magna* individuals after 24 h of exposure to different Zn^2+^ concentrations (without MPs) was not significantly different from the control (*p* (0.25 mg·L^−1^) = 0.2564; *p* (0.50 mg·L^−1^) = 1.000; *p* (1.00 mg·L^−1^) = 0.2794). On the contrary, significant differences at 95% confidence level were found in the presence of MPs, particularly at high Zn^2+^ concentrations. This trend is also more pronounced after 48 h, indicating that these microcontaminants affect the survival of *D. magna* individuals.

### Metal removal studies

Based on the similar outcomes observed for 0.5 and 1.0 mg·L^−1^ of Zn^2+^ in section “Effect of Zn^2+^ concentration,” a concentration of 0.5 mg·L^−1^ was used to evaluate the Zn^2+^ removal efficiency by *D. magna* in the presence of MP with different properties and aqueous matrices.

It is important to note that in the experiments presented in the following sections Zn^2+^ content measured in the organisms after the exposure period was lower than the value expected based on the percentage of Zn^2+^ removed from the solution.

This discrepancy may be attributed to the practical limitations associated with handling living organisms of such small size. Moreover, after 48 h of exposure, some individuals had died and it was difficult to fully recover them for the subsequent digestion analysis. Therefore, part of the Zn^2+^ removed from solution may have remained associated with unrecovered biomass.

#### Effect of MP properties

##### Commercial versus true-to-life MPs

Commercial MPs are manufactured with a uniform spherical shape and high polymer purity. In contrast, MPs derived from consumer plastics (true-to-life MPs) exhibit irregular shapes because their morphology cannot be controlled during their production and may contain other substances depending on the original plastic’s functional requirements. In Fig. S1, images of the commercial PE (63–53 μm) and true-to-life PE (80–63 μm) MPs were obtained with a portable USB digital microscope with LED illumination (resolution, 1600 × 1200 pixels; magnification, 1000×). Existing literature indicates that MP shape influences both the environmental fate of contaminants and the ecotoxicological effects on living organisms [26, 27]. Therefore, a 48 h exposure was performed to evaluate the impact of commercial and true-to-life MPs on *D. magna* mortality and Zn^2+^ removal efficiency. For this purpose, the mineral water containing 0.05% surfactant was spiked with 0.5 mg·L^−1^ Zn^2+^ and amended with either commercial or true-to-life PE MPs of similar size range or without them (Experiments 3 and 5, Table 1). A control without both Zn^2+^ and MPs was also conducted for comparison purposes (Control, MW, Table 1).

Results showed that MP shape does not influence Zn^2+^ removal from mineral water by *D. magna*, as can be seen in Table 3.

**Table 3:** Zn^2+^ adsorbed onto the microplastic and *D. magna* Zn^2+^ removal efficiency from the mineral water containing commercial and true-to-life polyethylene (PE) microplastics. Results expressed as mean ± standard deviation (*n* = 3)

	**Zn**^**2+**^** adsorption (%)**	**Zn**^**2+**^** removal efficiency (%)**
**MP type**	MP*	D+MP*
Commercial PE (63–53 µm)	n.d.	76 ± 2
True-to-life PE (80–63 µm)	n.d.	73 ± 1

*MP concentration: 100 mg·L^−1^ (15 mg/150 mL)

Nomenclature: *MP*, microplastic; *D+MP*, *Daphnia magna*+microplastic; *n.d.*, not detected

**Fig. 3:**
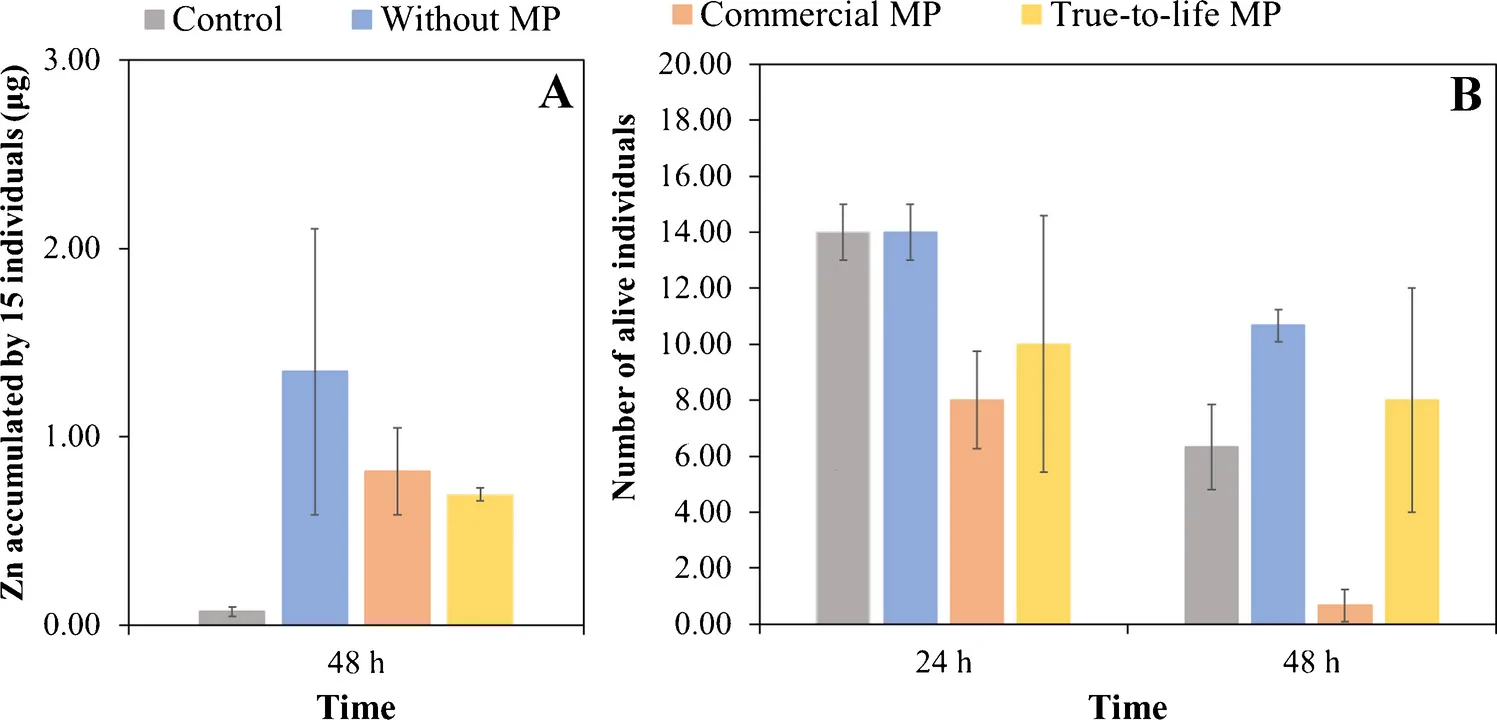
Zn^2+^ accumulated in *Daphnia magna* individuals (**A**) and *Daphnia magna* individuals’ survival (**B**) in the presence of commercial and true-to-life polyethylene microplastics. Experimental details can be found in Table 1 (Experiments Control, 3 and 5). Results are expressed as mean values of three independent replicates with the associated standard deviation

The same trend was observed in the results obtained from the Zn^2+^accumulation and the mortality of the living organisms. As can be seen in Fig. 3A, the content of Zn^2+^ within *D. magna* individuals was significantly higher than the control either when they were exposed to commercial or true-to-life MPs or even without the presence of MP. However, no significant differences in accumulation were observed between the two MP types (*t*-test, *p* > 0.05). Mortality rates were also similar when living organisms were exposed to both types of MP, becoming more pronounced after 48 h of exposure (see Fig. 3B). Therefore, the shape of the tested MPs did not influence Zn^2+^ removal by *D. magna*. Consequently, subsequent experiments were carried out with true-to-life MPs to better reflect environmental conditions.

##### Polymer type and aging process

The type of MP polymer can affect the metal adsorption process [28–30], and thus the ability of *D. magna* to eliminate Zn^2+^ may be influenced. Furthermore, several studies have pointed out the significant modifications MP surfaces may undergo when exposed to UV radiation [31–35]. To test the impact of these parameters on Zn^2+^ removal efficiency and the mortality of aquatic organisms, the individuals were exposed for 48 h to a Zn^2+^ solution (0.5 mg·L^−1^) and non-aged and aged true-to-life MPs of different materials (PE, PP, and PS) of the same size (63–80 μm) (Experiments 5, 6, 7, 9, 10, and 11; Table 1). As can be seen also in Table 1, the same experiments were also conducted involving both MPs and Zn^2+^ without *D. magna*, to assess the potential interactions between these pollutants.

The aged true-to-life MPs were produced by submitting the non-aged MPs of different polymers to an aging process consisting of UV radiation for 8 weeks at 42 °C, to simulate environmental degradation conditions associated with solar radiation exposure. To study more deeply the surface modifications caused by UV aging process, original and aged PE, PS and PP true-to-life MPs were characterized by FTIR. In Fig. S2 (see Electronic Supplementary Material), obtained IR spectra are displayed. As it is shown, UV aging of true-to-life PE, PS, and PP MPs modifies their surface chemistry by introducing oxygen-containing functional groups (mostly C=O), which can serve as specific binding sites for metal adsorption. The so-called Carbonyl Index (CI), widely used to monitor oxidation reactions in polymers, was also estimated for each type of MP. CI is calculated as the ratio of the carbonyl absorption peak with a reference peak, providing a quantitative measure of oxidative degradation and the progression of polymer aging [36]. Estimated CI values for non-aged MPs ranged from 0.11 to 0.69 and for UV-aged MPs from 1.03 to 2.11. As shown, significantly higher CI values were observed for UV-aged MPs across all polymer types, confirming oxidative degradation of the MP surface. It is also interesting to remark that the obtained CI values for the aged MPs were slightly higher but comparable with those obtained in real MP samples collected in different environmental scenarios [37].

Results displayed in Table 4 demonstrated that the percentage of Zn^2+^ adsorbed onto the studied non-aged MPs was below 10% for the three polymers studied. This reveals that the polymer type does not influence non-aged MP-metal interaction during the experiment. Godoy et al. [38] demonstrated that Zn^2+^ adsorption equilibrium onto different MP materials was typically reached within 5 days, suggesting that the experimental duration in this work was most likely not long enough for the metal to be completely adsorbed onto the MPs. However, the Zn^2+^ adsorption onto PE MPs and PP MPs increased with aging to 27% and 8%, respectively. It is known that aging causes physical and chemical alteration on the surfaces of MPs, such as an increase in roughness and the presence of oxygen-containing functional groups, which can act as binding sites for Zn^2+^ ions, thereby enhancing their adsorption [14]. This implies that, if a *D. magna* reactor is constructed in a wastewater treatment plant as tertiary or quaternary treatment step in a sunny region, then the MPs will be prone to undergo aging due to solar radiation, which will enhance their interaction with metals.

**Table 4:** Zn^2+^ adsorbed onto the microplastic and the *D. magna* Zn^2+^ removal efficiency from mineral water containing true-to-life microplastics of different materials (*PE*, polyethylene; *PP*, polypropylene; *PS*, polystyrene). Results expressed as mean ± standard deviation (*n* = 3)

	**Zn**^**2+**^** adsorbed (%)**	**Zn**^**2+**^** removal efficiency (%)**
**MP type**	MP*	D+MP*
Non-aged PE (80–63 µm)	n.d.	73 ± 1
Aged PE (80–63 µm)	27 ± 3	71 ± 8
Non-aged PP (80–63 µm)	2.6 ± 0.2	83 ± 6
Aged PP (80–63 µm)	8.3 ± 0.8	76 ± 7
Non-aged PS (80–63 µm)	4.8 ± 0.1	55 ± 13
Aged PS (80–63 µm)	n.d.	72 ± 22

*MP concentration: 100 mg·L^−1^ (15 mg/150 mL)

Nomenclature: *MP*, microplastic;*D+MP*, *Daphnia magna*+microplastic; *n.d.*, not detected

In the presence of *D. magna* individuals, no significant differences (*p* > 0.05) were found in the Zn^2+^ removed from the aqueous solution containing the non-aged MPs under study after 48 h of exposure, except for PS MP, for which the Zn^2+^ elimination rate was lower (55 ± 13%) than that determined for the other MPs (PE = 73 ± 1%; PP = 83 ± 6%). Although the MP aging seemed to slightly affect the aged MP-metal interaction, *D. magna* individuals removed a similar quantity of Zn^2+^ from the water in the presence of the different aged MPs, achieving removal efficiencies between 71 and 76% (see Table 4).

**Fig. 4:**
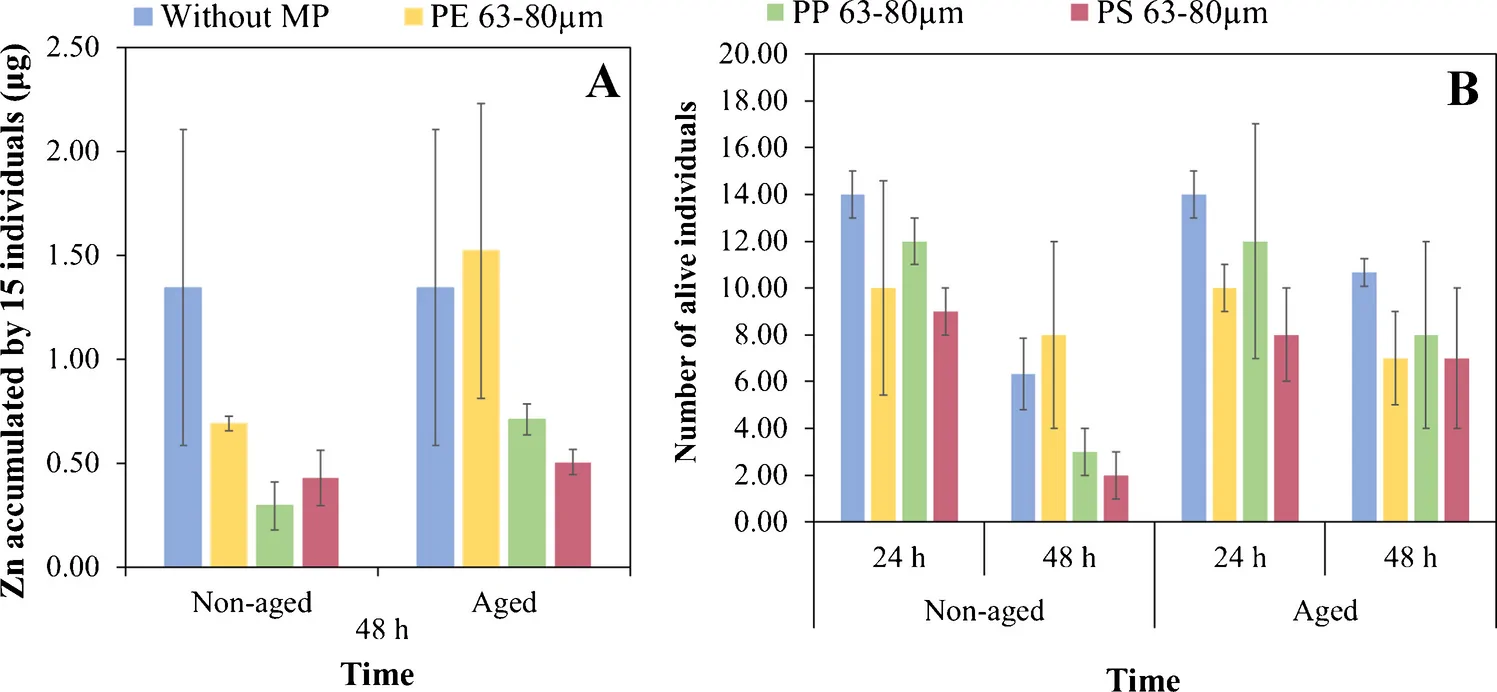
Zn^2+^accumulated in *Daphnia magna* individuals (**A**) and *Daphnia magna* individuals’ survival (**B**) in the presence of non-aged and aged microplastics of different materials. Acronyms: PE, polyethylene; PP, polypropylene; PS, polystyrene. Experimental details can be found in Table 1 (Experiments 3, 5, 6, 7, 9, 10, 11, and 12). Results are expressed as mean values of three independent replicates with the associated standard deviation

The amount of Zn^2+^ accumulated by the individuals was slightly higher in the presence of PE MPs compared to PP and PS MPs, particularly in the case of UV-aged PE MPs (Fig. 4A). As shown in Table 5, this type of MPs also exhibited the highest Zn^2+^ adsorption capacity. Therefore, it appears that metal accumulation in *D. magna* increases when the metal is adsorbed onto MPs. However, further studies are needed to confirm this trend. The elevated Zn^2+^ levels in *D. magna* individuals exposed to PE MPs suggest that these particles act as a more efficient vector for metal transport than PP and PS MPs. This correlated with the higher adsorption capacity of PE MPs shown in Table 5, indicating that the ingestion of PE MPs significantly increases total Zn^2+^ accumulation in *D. magna* individuals. On the other hand, as it is shown in Fig. 4B, MP exposure increased *D. magna* mortality, particularly after 48 h, irrespective of MP type.

**Table 5:** Zn^2+^ adsorbed onto the microplastic and the *D. magna* Zn^2+^ removal efficiency from mineral water containing true-to-life polypropylene (PP) microplastics of different sizes. Results expressed as mean ± standard deviation (*n* = 3)

	**Zn**^**2+**^** adsorbed (%)**	**Zn**^**2+**^** removal efficiency (%)**
**MP type**	MP*	D+MP*
PP (< 63 µm)	14.4 ± 0.9	81 ± 1
PP (80–63 µm)	3 ± 1	83 ± 6

*MP concentration: 100 mg·L^−1^ (15 mg/150 mL)

Nomenclature: *MP*, microplastic; *D+MP*, Daphnia magna+microplastic

##### MP size

MP size can play a critical role in their interactions with metals and their ingestion by aquatic organisms. Smaller particles can be easily ingested by species such as *D. magna*, whereas larger particles may exert different effects, including enhanced contaminant adsorption or mechanical obstruction within the digestive tract [13]. These differences may substantially influence the survival of these aquatic species and their capacity to accumulate metals such as Zn^2+^.

To evaluate the impact of MP size on *D. magna* Zn^2+^ filtration capacity and mortality, the individuals were exposed for 48 h to a Zn^2+^ solution (0.5 mg·L^−1^) and true-to-life PP MPs of two size fractions (< 63 μm and 63–80 μm) (Experiments 7–8, Table 1). Experiments involving both Zn^2+^ and MPs, without the presence of *D. magna*, were also performed to evaluate potential interactions between these two contaminants.

Results showed that smaller MPs adsorbed 14% of the Zn^2+^ present in the aqueous solution, meanwhile larger MPs only adsorbed 3% (see Table 5). These findings are consistent with those of Nguyen et al. [39], who reported that the Pb^2+^ adsorption capacity of PE MPs increased significantly with decreasing the MP particle size. Similarly, Soheilian et al. [40] found that smaller PS MPs are more prone to adsorb Cu^2+^ than larger plastic particles. Muthuraja et al. [41] also demonstrated that smaller MPs, regardless of their composition (PE, PS), adsorb more metals (Cu^2+^, Pb^2+^) than larger MPs. In the presence of *D. magna*, Zn^2+^ elimination rates ranged 81–83%, with no significant differences (*p* > 0.05) observed between the presence of different sized PP MPs (< 63 μm and 63–80 μm). Despite the similar Zn^2+^ removal efficiencies in the presence of both MP size ranges, further investigations will be conducted in the future to verify whether various MP sizes below 63 μm affect the ingestion of Zn^2+^ by *D. magna* in a similar manner.

**Fig. 5:**
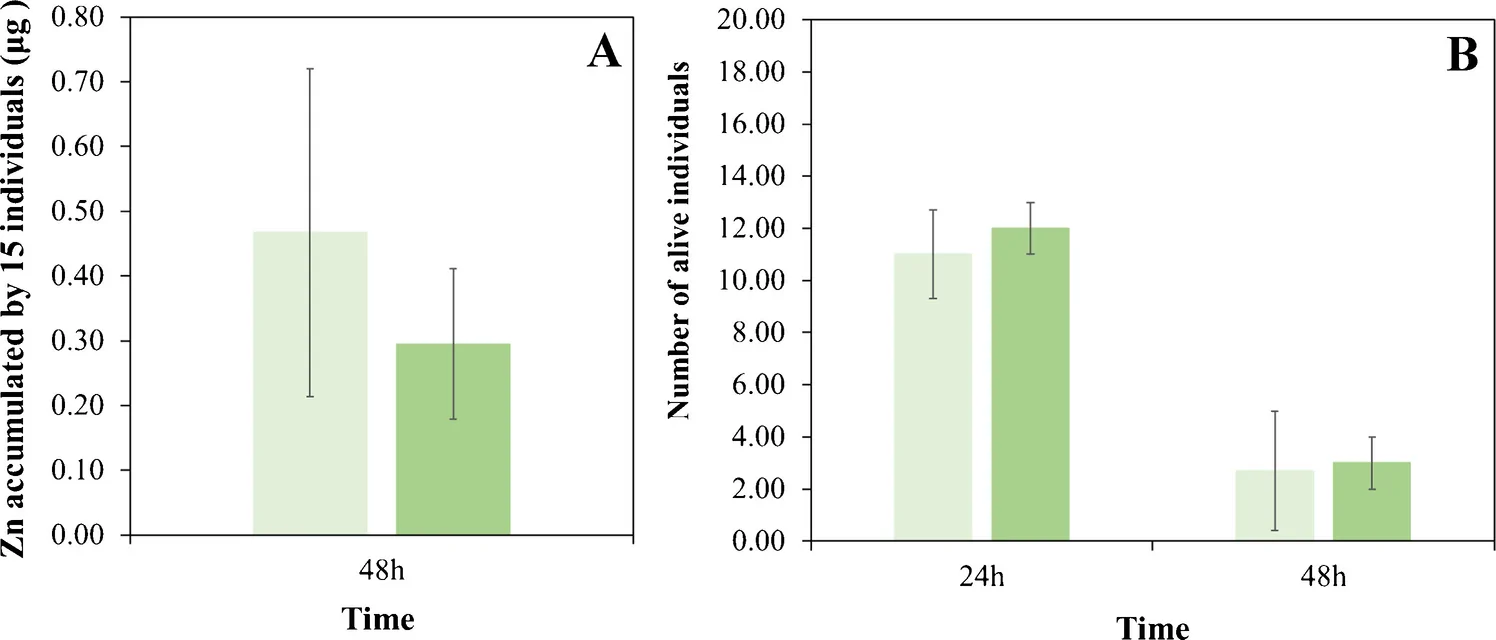
Zn^2+^ accumulated in *Daphnia magna* individuals (**A**) and *Daphnia magna* individuals’ survival (**B**) in the presence of different sized polypropylene (PP) microplastics. Experimental details can be found in Table 1 (Experiments 3, 7, and 8). Results are expressed as mean values of three independent replicates with the associated standard deviation

As can be seen in Fig. 5A, the metal accumulated by these aquatic organisms was not significantly different in the presence of the MPs used in this experiment (*p* > 0.05). These results suggest that the filtration capacity of *D. magna* for Zn^2+^ is not influenced by the MP size under the experimental conditions employed. Mortality rates were similar between the different size fractions of PP MPs (< 63 μm and 63–80 μm), with a significant increase observed after 48 h (see Fig. 5B).

##### Aqueous matrix

As mentioned in section “Water samples,” the influence of the water matrix on the filtration capacity of *D. magna* for Zn^2+^ was evaluated with mineral water and wastewater from a pilot scale *Daphnia* treatment system. Additional information about the chemical composition of these waters is displayed in Table S1 (see Electronic Supplementary Material).

Experiments were carried out by spiking the waters, containing 0.05% surfactant, with 0.5 mg·L^−1^ of Zn^2+^. The effect of the presence of MPs was studied adding to selected water samples non-aged and aged PP MPs of 63–80 µm (Experiments 7, 9, 12, and 13, Table 1). The same experiments were also conducted involving both MPs and Zn^2+^ but without *D. magna*, to assess the potential interactions between these contaminants in both aqueous matrices. Results showed that even though the Zn^2+^ adsorption onto PP MPs was higher in wastewater, this fact does not influence the Zn^2+^ removal efficiency by *D. magna* (see Table 6).

**Table 6:** Zn^2+^ adsorbed onto the MP and *D. magna* Zn^2+^ removal efficiency from mineral water or wastewater containing true-to-life non-aged or aged polypropylene (PP) microplastics. Results expressed as mean ± standard deviation (*n* = 3)

		**Zn**^**2+**^** adsorbed (%)**	**Zn**^**2+**^** removal efficiency (%)**
	**MP type**^a^	MP*	D+MP*
**Mineral water**	Non-aged PP (80–63 µm)	3 ± 1	76 ± 2
	Aged PP (80–63 µm)	8.3 ± 0.8	76 ± 3
**Wastewater**	Non-aged PP (80–63 µm)	12.6 ± 0.7	72 ± 1
	Aged PP (80–63 µm)	12 ± 5	71.2 ± 0.5

*MP concentration: 100 mg·L^−1^ (15 mg/150 mL)

Nomenclature: *MP*, microplastic; *D+MP*, *Daphnia magna*+microplastic

**Fig. 6:**
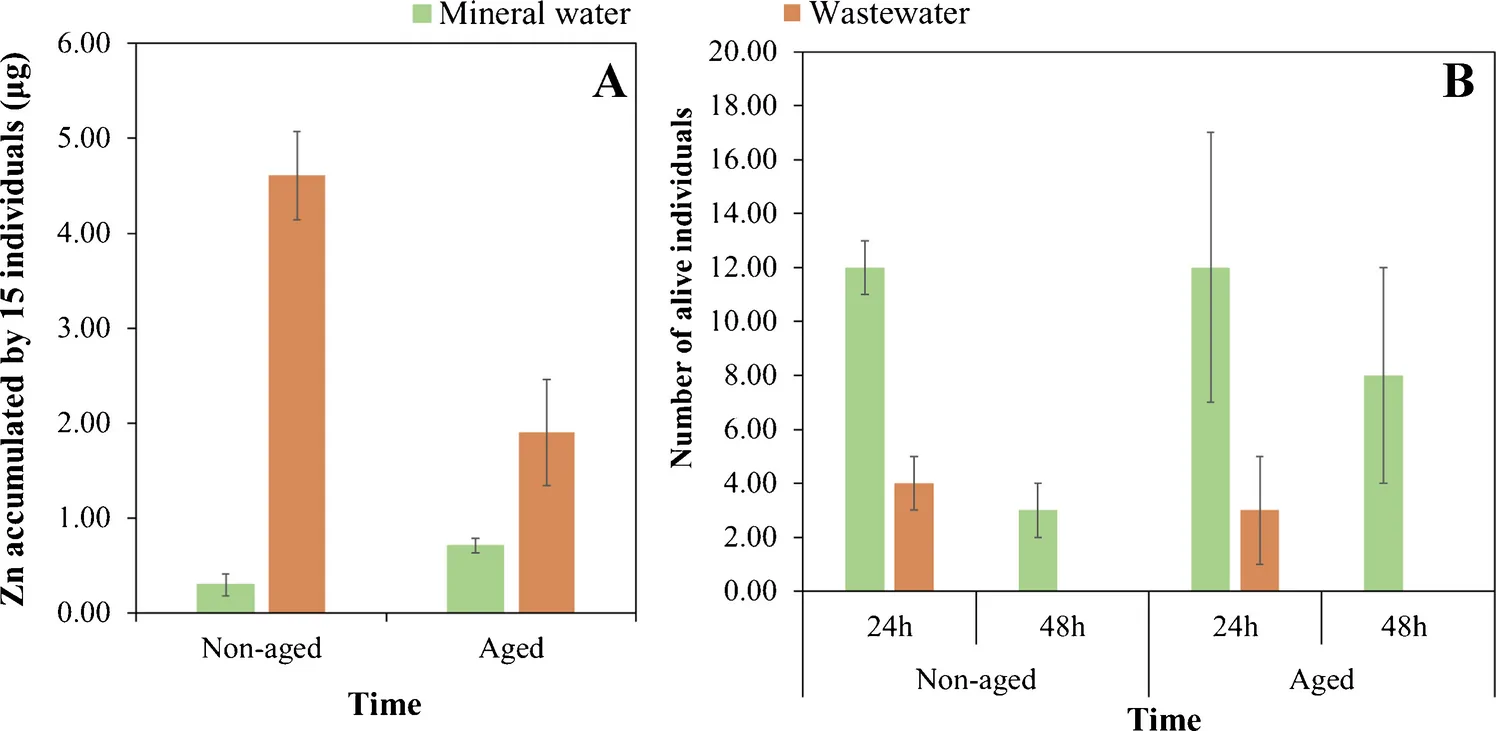
Zn^2+^accumulated in *Daphnia magna* individuals (**A**) and *Daphnia magna* individuals’ survival (**B**) in mineral water and wastewater matrix. Experimental details can be found in Table 1 (Experiments 7, 9, 12, and 13). Results are expressed as mean values of three independent replicates with the associated standard deviation

Zn^2+^ accumulation in *D. magna* was significantly higher (*t*-test, *p* < 0.05) in wastewater compared to mineral water for both types of PP MPs tested (Fig. 6A). This increase may be attributable to the higher concentration of suspended solids in the media (see Table S1 from the Electronic Supplementary Material), which can adsorb the metal and enhance its accumulation in *D. magna* following ingestion of the metal-laden particles. Mortality was also higher in wastewater; however, it remained comparable between 24 and 48 h for both non-aged and aged MPs.

## Conclusions

In this contribution, the ability of *D. magna* to remove Zn^2+^ from the aqueous medium, both in the presence and absence of MPs, was demonstrated. Although in some cases Zn^2+^ was partially adsorbed onto MPs, rather than remaining in solution, this did not affect the metal removal efficiency by *D. magna*, which remained consistently around 80%. The ingestion and accumulation of MPs in *D. magna*’s digestive tract was also verified, showing *D. magna*’s capacity to remove such type of microparticulate emerging pollutants from waters.

In general, the presence of both commercial and true-to-life MPs did not significantly affect Zn^2+^ removal from water by *D. magna*, although MP exposure increased mortality after 24–48 h. MP polymer type and aging influenced Zn^2+^ bioaccumulation, with slightly higher accumulation observed in the presence of PE MPs compared to PP and PS. MP size had a negligible effect on Zn^2+^ removal, at least within the studied range (< 63 vs. 63–80 µm). Results also demonstrated that the composition of the aqueous media strongly influences Zn^2+^ bioaccumulation in the living organisms. In wastewater, it was significantly higher than in mineral water, likely due to metal adsorption onto suspended solids, which enhances its uptake by *D. magna* via ingestion of the metal-laden particles.

These findings highlight the potential of *D. magna*–based eco-sustainable wastewater treatment systems for the removal of micropollutants, including metals and MPs. Future studies will evaluate metal removal efficiency across additional wastewater matrices and with other MP types, including biodegradable polymers.

## Supplementary Information

Below is the link to the electronic supplementary material.

Supplementary file1 (PDF 376 KB)

## Data Availability

The data from the experiments presented throughout this research paper may be obtained from the authors upon reasonable request.
