# Supplementary material for: Influence of aged and non-aged environmentally relevant microplastics on zinc removal from aqueous media by Daphnia magna
Source: Anal Bioanal Chem. 2026 Jun 12;418(18):6129–40. doi: 10.1007/s00216-026-06592-1 (PMC13541781; doi:10.1007/s00216-026-06592-1)
Supplement: Supplementary file 1 [file 216_2026_6592_MOESM1_ESM.pdf]

SUPPLEMENTARY MATERIAL

Influence of aged and non-aged environmentally relevant microplastics on zinc removal from aqueous media by Daphnia magna

Mary Isabel Villacís[1], Laura Torrent[1,*], Mònica Iglesias[1], Emili Besalú[1], Laura Borgese[2], Stefania Federcici[2], Eva Marguí[1,*]

[1] Department of Chemistry, University of Girona, Girona, Spain

[2] Department of Mechanical and Industrial Engineering, University of Brescia, Brescia, Italy

Table S1. Chemical characterization of the mineral water and wastewater samples used in *D. magna* experiments.

Parameter	Units	Mineral water	Wastewater
pH	-	7.34	6.99
Solid suspension	$mg{\cdot}L^{-1}$	0	1.5
Turbidity	UNT	0	3.04
COD	$mg\ O_2{\cdot}L^{-1}$	<DL	27
Conductivity	µS/cm	258	1246
$N\text{-}NO_2^-$	$mg{\cdot}L^{-1}$	<DL	0.555
$N\text{-}NO_3^-$	$mg{\cdot}L^{-1}$	0.3	0.581
$N\text{-}PO_4^{3-}$	$mg{\cdot}L^{-1}$	<DL	2.933
$N\text{-}NH_4^+$	$mg{\cdot}L^{-1}$	<DL	25.32
B	$mg{\cdot}L^{-1}$	<0.25	0.413 ± 0.002
Ca	$mg{\cdot}L^{-1}$	45 ± 3	74.1 ± 0.6
Fe	$mg{\cdot}L^{-1}$	0.69 ± 0.05	157 ± 2
K	$mg{\cdot}L^{-1}$	1.62 ± 0.07	43.8 ± 0.6
Mg	$mg{\cdot}L^{-1}$	10.8 ± 0.6	14.82 ± 0.07
Mo	$mg{\cdot}L^{-1}$	0.084 ± 0.003	<0.06
Na	$mg{\cdot}L^{-1}$	17.5 ± 0.9	194 ± 9
V	$mg{\cdot}L^{-1}$	<0.06	<0.06
Ag	$\mu g{\cdot}L^{-1}$	<0.2	<0.2
As	$\mu g{\cdot}L^{-1}$	<0.2	0.6 ± 0.3
Cd	$\mu g{\cdot}L^{-1}$	<0.05	<0.05
Co	$\mu g{\cdot}L^{-1}$	0.069 ± 0.004	0.56 ± 0.02
Cr	$\mu g{\cdot}L^{-1}$	<0.05	0.47 ± 0.08
Cu	$\mu g{\cdot}L^{-1}$	<0.2	1.92 ± 0.09
Mn	$\mu g{\cdot}L^{-1}$	<1	18 ± 2
Ni	$\mu g{\cdot}L^{-1}$	<0.2	2.18 ± 0.04
Pb	$\mu g{\cdot}L^{-1}$	0.17 ± 0.04	0.16 ± 0.02
Se	$\mu g{\cdot}L^{-1}$	0.5 ± 0.2	0.4 ± 0.2
Ti	$\mu g{\cdot}L^{-1}$	1.1 ± 0.1	4.5 ± 0.6
Zn	$\mu g{\cdot}L^{-1}$	3.3 ± 0.2	13.7 ± 0.6

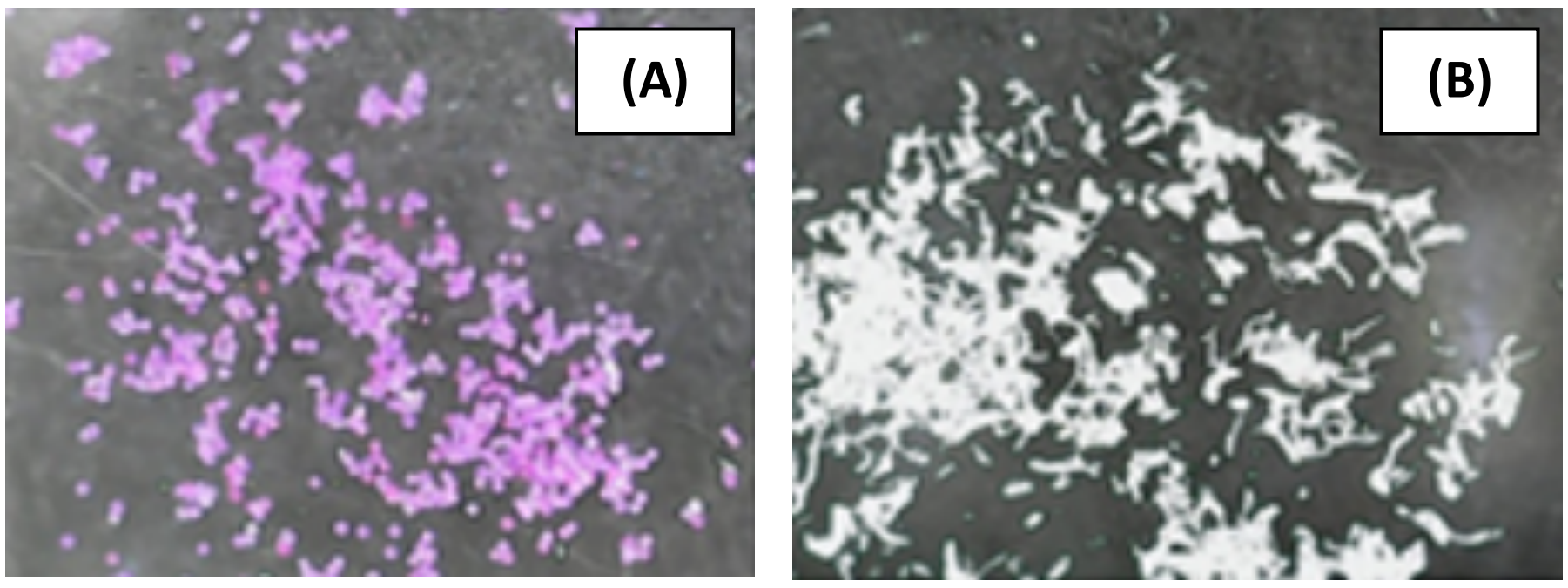

Fig. S1. Images of the: (A) commercial PE (63-53 μm) and (B) true-to-life PE (80-63μm) MPs obtained with a portable USB digital microscope with LED illumination (Resolution: 1600x1200 pixels, magnification: 1000x).

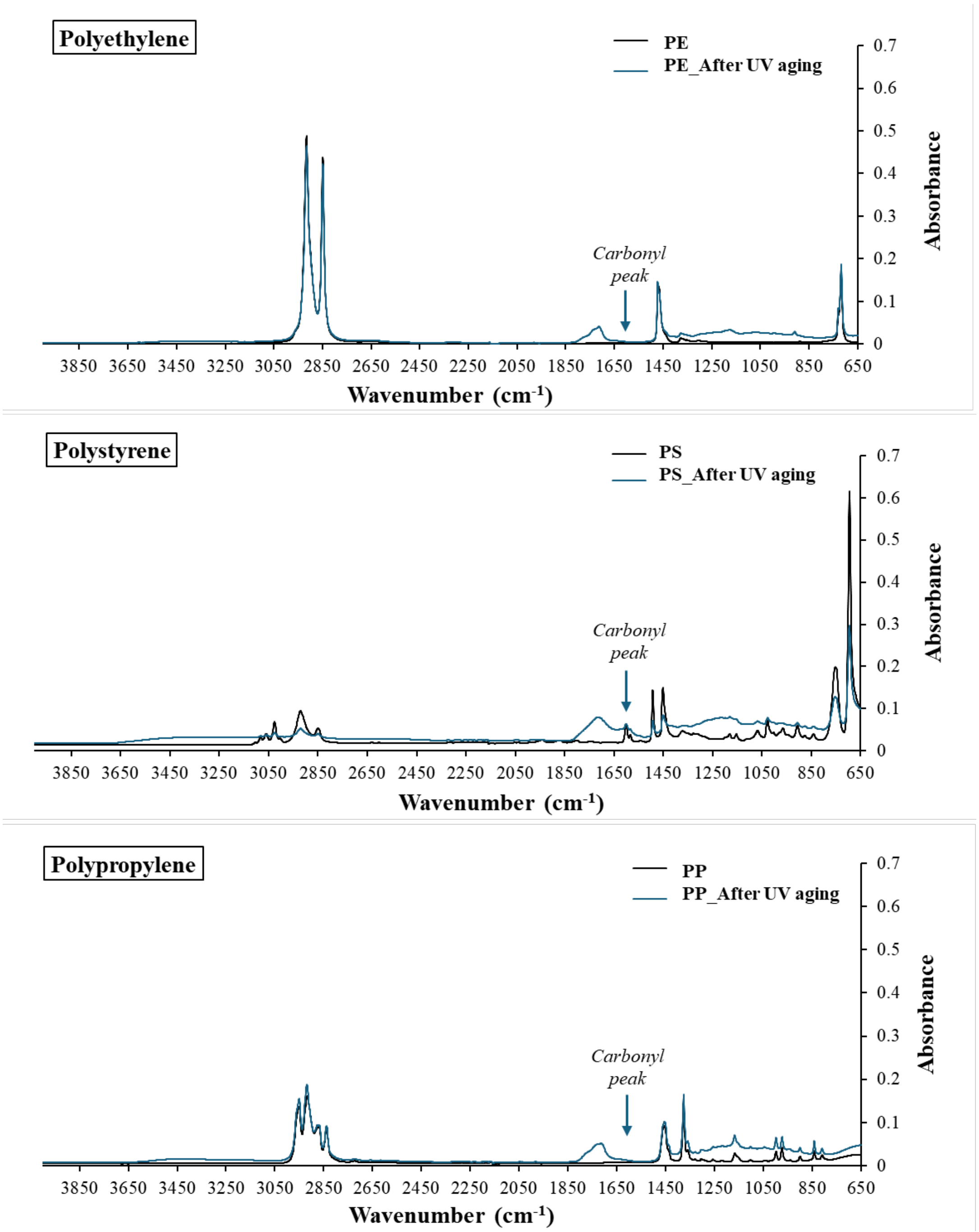

Fig. S2. IR spectra obtained for pristine and UV aged “true-to-life” MPs (63-80 µm).
